# A Pilot Study on the Impact of the BumptUp^®^ Mobile App on Physical Activity during and after Pregnancy

**DOI:** 10.3390/su141912801

**Published:** 2022-10-07

**Authors:** Rachel A. Tinius, Maire M. Blankenship, Alison M. Colao, Gregory S. Hawk, Madhawa Perera, Nancy E. Schoenberg

**Affiliations:** 1 Exercise Science, Western Kentucky University, Bowling Green, KY 42101, USA; 2 Nursing and Allied Health, Western Kentucky University, Bowling Green, KY 42101, USA; 3 Department of Statistics, University of Kentucky, Lexington, KY 40506, USA; 4 Gender and Women’s Studies, College of Arts and Sciences, University of Kentucky, Lexington, KY 40506, USA

**Keywords:** pregnancy, postpartum, exercise, mhealth

## Abstract

To combat maternal morbidity and mortality, interventions designed to increase physical activity levels during and after pregnancy are needed. Mobile phone-based interventions show considerable promise, and BumptUp^®^ has been carefully developed to address the lack of exercise among pregnant and postpartum women. The primary goal of this pilot study was to test the potential efficacy of BumptUp^®^ for improving physical activity among pregnant and postpartum women. A randomized controlled clinical trial was performed (N = 35) with women either receiving access to the mhealth app or an educational brochure. Physical activity and self-efficacy for exercise data were collected at baseline (in mid-pregnancy) and at three additional timepoints (late pregnancy, 6 and 12 weeks postpartum). For moderate-to-vigorous physical activity, a clear trend is observed as the mean estimated difference between groups increases from −0.35 (SE: 1.75) in mid-pregnancy to −0.81 (SE: 1.75) in late pregnancy. For self-efficacy for exercise, the estimated difference of means (control–intervention) changed from 0.96 (SE: 6.53) at baseline to −7.64 (SE: 6.66) in late pregnancy and remained at −6.41 (SE: 6.79) and −6.70 (SE: 6.96) at 6 and 12 weeks postpartum, respectively. When assessing the change in self-efficacy from mid-to -ate pregnancy only, there was a statistically significant difference between groups (*p* = 0.044). BumptUp^®^ (version 1.0 (3)) shows potential for efficacy. Pilot data suggest key refinements to be made and a larger clinical trial is warranted.

## 1. Introduction

Maternal mortality and morbidity rates are strikingly high in the U.S. compared to other industrialized countries [1]. A contributing factor is the large number of women presenting for prenatal care with chronic health conditions including obesity, hypertension, and diabetes, all of which lead to complicated pregnancies [2], and women in certain marginalized groups may be even more vulnerable to chronic health conditions and have less access to prenatal care than women in other settings [3,4].

Physical activity during and after pregnancy contributes to better/optimal maternal health outcomes including reduced weight gain, improved glucose control, lower blood pressure, and better mental health [5]. Despite these well-documented findings, there are few sustainable, scalable, and accessible interventions that can successfully increase physical activity and reduce gestational weight gain, particularly among underserved women.

Mobile phone-based interventions show considerable promise because they can be tailored to the target population, can be delivered at any place and at any time, are interactive, and are accessible to the majority of the population irrespective of socioeconomic status (96% of U.S. women aged 18–49 have smartphones) [6,7]. Previous research demonstrates the ability of mobile apps to positively impact physical activity behaviors [8]; thus, a unique and timely opportunity exists to increase physical activity among pregnant and postpartum women through mobile health apps.

Mobile apps have emerged as a primary mode of health information for women during pregnancy [9]. In fact, pregnancy is the medical condition with the highest number of apps available [9]. However, to our knowledge, mobile apps designed specifically to increase physical activity during pregnancy are sparse [8,10,11]. Mobile apps designed to alter lifestyle during pregnancy have been unable to elicit statistically significant differences in physical activity among intervention vs. control groups of pregnant women [12–14]. In addition, many focus more on weight status and diet than physical activity [12,13,15]. Ainscough et al. focused on the impact of a mobile health program for overweight and obese pregnant women and found some improvements among physical activity variables in the intervention arm [15]; however, the intervention contained limited physical activity guidance (i.e., no personalized programming) and physical activity was measured via self-report, which is a major limitation. Several recent reviews concluded that exercise apps designed for pregnancy fail to consider: (1) current evidence-based physical activity guidelines, (2) screening for contraindications to physical activity, (3) appropriate personalization features to account for an individual’s characteristics, and (4) the involvement of qualified experts during the development of the app [10,11].

Akin to the postpartum focus on infant care rather than maternal health [16], a limitation to nearly all of the aforementioned mobile apps is the lack of continuation of physical activity support during the postpartum period [8]. Over 50% of maternal deaths occur between 7 and 365 days postpartum, many of which are related to factors modifiable with physical activity (i.e., obesity, diabetes, hypertension, and mental health) [4]. Given that pregnancy is an important opportunity for making lasting lifestyle changes [17], and keeping women engaged through postpartum can help with long-term adherence, the development of an mHealth app that also assists women through the postpartum period is critical.

To address these gaps, our team has engaged in extensive preliminary studies including focus groups and interviews with pregnant women, postpartum women, and obstetric health care providers [18] in order to develop an acceptable and promising approach to increase physical activities during and after pregnancy. Key features identified from these sessions and integrated into the development of a mobile health app (BumptUp^®^) include progress tracking, social support, evidence-based and safe exercise programming, videos, and symptom tracking [18], and these features set apart BumptUp^®^ from existing mobile health interventions for pregnant and postpartum women. The primary goal of this pilot study was to test the efficacy of BumptUp^®^ for improving physical activity and self-efficacy for physical activity among pregnant and postpartum women.

## 2. Materials and Methods

Participants: Study information was provided via social media, the physicians at the designated health clinic, and word of mouth. Sixty-nine women reached out regarding participation between November 2020 and August 2021. Inclusion criteria included: (1) Age 18–44; (2) Confirmed singleton viable pregnancy; (3) English-speaking (the app is currently only available in English); (4) Physician release to participate in exercise; (5) Ownership and willingness to use a smartphone; and (6) Plans to deliver at The Medical Center in Bowling Green, KY. Exclusion criteria included: (1) Multiple gestation pregnancy; (2) Inability to provide voluntary informed consent; and (3) Any medical condition (pregnancy-related or not) that would preclude exercise. All 69 were screened; 38 were deemed eligible and enrolled. Three women dropped out (one experienced a spontaneous abortion (control group) and two were lost to follow up (intervention)); thus, a total of 35 were included in the final analyses.

**Figure 1.**
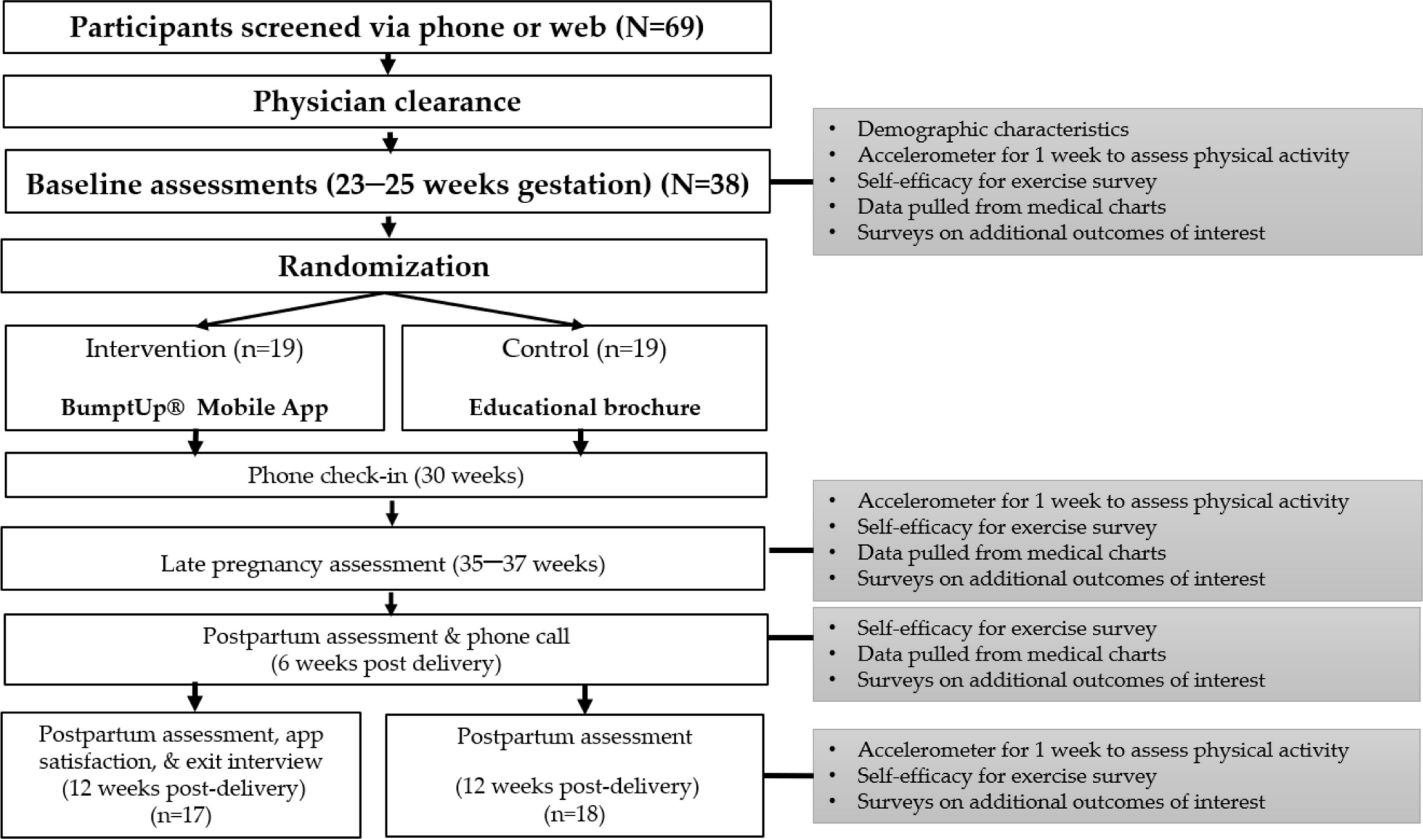
Study flowchart and procedures.

Study procedures: All procedures were approved by the Western Kentucky University Institutional Review Board (#20–257) and registered with clinicaltrials.gov (NCT04480931). An overview of the study design is provided in Figure 1.

Data were collected at the following timepoints: baseline/mid-pregnancy (23–25 weeks), late pregnancy (35–37 weeks), and 6 and 12 weeks postpartum. Of note, 12-week interventions during pregnancy are customary and have been shown to elicit clinically meaningful changes in outcomes [19,20]. There were two telephone check-ins (one during pregnancy and one during postpartum upon medical clearance at ~6 weeks) to aid with retention, as well as allow participants to ask any questions about the app or the educational brochure. The late-pregnancy timepoint was selected as women tend to be least active during late pregnancy [21]. Six weeks postpartum was chosen, as this is when most women have their only postpartum clinical appointment (and thus presented an opportunity for objective clinical data extraction). The 12-week time point was selected for assessing behavior as women transition back into activity after medical clearance during the early postpartum days, which is important for long-term sustained activity.

After the baseline assessment (~23–25 weeks gestation), participants were randomized into the intervention or control group. The intervention group received free access to the mobile app (BumptUp^®^). BumptUp^®^ users were started with an introductory video on the app’s features which reiterated the goal of the app: to reach the recommended 150 min per week of physical activity as per recommendations from the American College of Obstetricians and Gynecologists [5]. The app contains evidence-based workout information and education as well as several resources aimed at increasing physical activity and overcoming barriers for women during and after pregnancy. Key additional features of the app include physical activity tracking, customized calorie tracking, customized gestational weight gain tracking with visuals, pregnancy-specific exercise videos with modifications and difficulty ranges, a build-your-own-workout system, symptom-tracking with a flagging feature for when to contact a provider, social support and communication, weekly educational articles, and careful screening mechanisms built into the flow of the app.

The control group underwent the exact same study protocols and received the same amount of attention from the study team (an attention control); however, control participants received an evidence-based educational brochure about physical activity during pregnancy instead of access to BumptUp^®^. The evidence-based brochure has been shown to increase knowledge and influence beliefs about physical activity during pregnancy [22], and an intervention utilizing the educational brochure was shown to reduce sedentary time during pregnancy [23].

Data collection: To assess the primary outcome of physical activity, participants wore an Actigraph wGT3X-BT Accelerometer (ActiGraph, LLC, Pensacola, FL, USA) on their wrist for seven consecutive days at each time point (Pregnancy: 23–25 weeks, 35–37 weeks, Postpartum: 12 weeks). Monitoring physical activity for one week at multiple time points is standard in physical activity research [24]. Wrist-worn tri-axel accelerometers are a valid measure of physical activity in pregnant women [25]. Devices were initialized by the study team in the Exercise Physiology Laboratory and delivered to each participant at a location of their choice. At the end of the 7 days, the study team picked up the device from the location desired by the participant. Data were collected for seven consecutive days at 30 Hz. The accelerometer output was sampled by a 12-bit analog-to-digital converter. Categories of activity were determined using the following cut points: Sedentary (0–99 counts/minute), light (100–1951 counts/min), moderate (1952–5724 counts/minute), and vigorous (≥5725 counts/min) [26]. The percentage of time spent sedentary as well as the amount of time spent participating in different categories of physical activity ranging from light to vigorous was calculated. Non-wear times were excluded from the analyses. Women were asked to wear the devices for 7 consecutive days without removal. If they did remove it, they were asked to document it so the study team could be sure it did not interfere with the analyses. The study coordinated with participants to select a week when they did not anticipate a reason for removal. As such, compliance was very high. All women wore the device for all 7 days with few or no removals.

Physical activity was also assessed subjectively using the Pregnancy Physical Activity Questionnaire (PPAQ) [27]. Self-efficacy was assessed via the Self-Efficacy for Physical Activity Survey [28] and delivered to the patient electronically via Research Electronic Data Capture (REDCap)(version 11.2.2) [29]. Self-efficacy was chosen as an important outcome to assess as self-efficacy is intricately linked to engagement in physical activity [30], and this is especially true among pregnant women [31,32].

Electronic surveys were distributed at each time point via REDCap [29]. Additional surveys which will serve as additional future outcomes and/or potential covariates in the larger trial included: General Demographics Survey, The Edinberg Postpartum Depression Questionnaire [33,34], Center for Epidemiological Studies Depression Scale [35], Social Support and Exercise Survey [36,37], CDC Barriers to being Active Quiz [38], Pregnancy Symptoms Inventory [39], Pelvic Floor Health (PDFI-I) [40], and the National Cancer Institute Multifactor (Diet)Screener [41]. Some surveys will be used to determine the potential impact of the mHealth intervention on a particular outcome related to maternal health. For example, mental health surveys (Edinberg Postpartum Depression Questionnaire and the Center for Epidemiological Studies Depression Scale) were included as depression plays a significant role in maternal morbidity and mortality [42,43], and physical activity improves depressive symptoms during and after pregnancy [44]. Therefore, it is useful to understand if the mHealth app improves mental health.

At the end of the protocol, participants assigned to the intervention group were given an app satisfaction survey (using the Mobile Application Rating Scale (MARS)) [45] in addition to one-on-one exit interviews. The MARS survey is a validated instrument and has excellent reliability (Omega 0.79 to 0.93) [46].

Medical Data from Participant Charts: Key medical data were collected directly from patient electronic medical record systems. These data included height, weight, and blood pressure values at prenatal appointments that correspond to data collection time points (i.e., 23–27 weeks-wider time range due to appointments still being monthly for many women during early-to-mid pregnancy, 35–37 weeks, and 6-weeks postpartum). All data at 12 weeks postpartum were patient/participant self-report as there are no routine postpartum visits at 12 weeks as per the standard-of-care. These extracted data were used to determine the impact of the intervention on weight gain, weight retention, blood pressure, and glucose tolerance.

Statistical Analysis: Pilot data are used to provide an estimate of the standard deviation and effect size, which will then be used to determine sample size and what will be observed in the main trial (forthcoming). Therefore, statistically significant differences between groups were not expected. However, appropriate statistical tests were still performed in order to examine trends and estimate effect sizes to use in the planning of a larger trial in the future.

The baseline characteristics of the intervention and control groups were compared using *t*-tests and chi-square tests, as appropriate. To investigate the effect of the intervention on study outcomes over time, a full-factorial repeated-measures ANCOVA model was fit for each outcome, analyzing overall differences across the two groups at each timepoint while adjusting for body mass index (BMI). Likelihood ratio testing and Akaike Information Criterion (AIC) were used to select appropriate covariance structures in each case. Due to the pilot nature of the study, group-level least-square means and their pairwise differences were calculated at each timepoint and adjusted for multiple comparisons, as appropriate. A Kenward–Roger adjustment was used to correct for negative bias in the standard errors and degrees of freedom calculations induced by the small sample size. All analyses were completed in SAS 9.4 (SAS Institute Inc.; Cary, NC, USA) or SPSS (version 28). All data were entered, stored, and maintained in the REDcap data management system [29].

## 3. Results

There were no baseline differences between the control and intervention groups for any of the demographic variables assessed including BMI, marital status, income level, race/ethnicity, educational attainment, employment status, self-reported health status, and physical activity levels. Table 1 contains demographic information for control and intervention participants.

The mean pre-pregnancy BMI of the study cohort was 29.0 ± 7.5 kg/m^2^ (range 21.4–59.4 kg/m^2^). In the study sample, 29% of participants had pre-pregnancy BMI within normal limits, while 38% were classified as overweight and 33% were classified as obese. According to the Centers for Disease Control, ~37% of women of childbearing age in Kentucky are classified as obese [47], suggesting a sample of women representative of women in the community.

**Figure 2.**
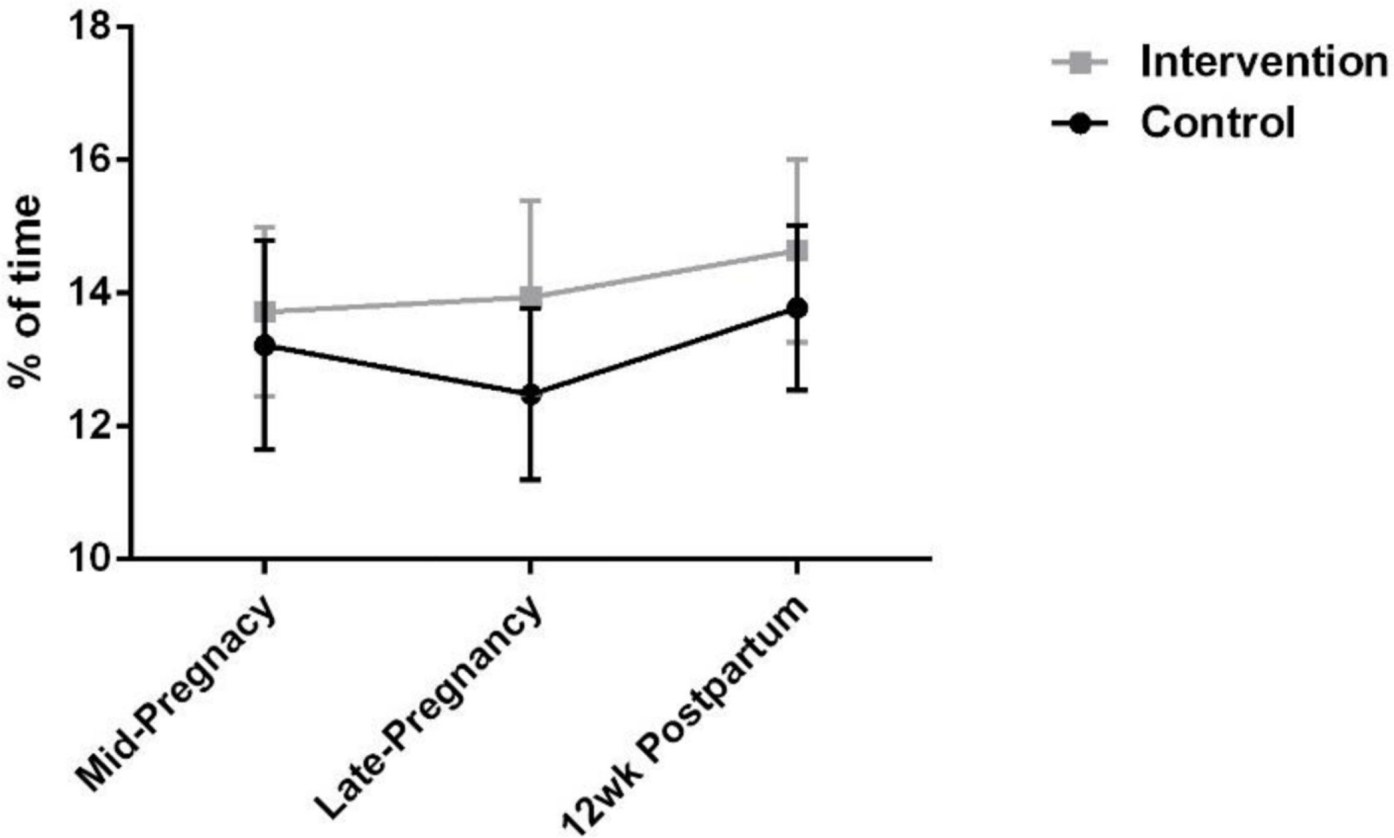
Moderate-to-vigorous physical activity levels between control and intervention participants (mean ± SEM).

Efficacy: There were no statistically significant differences between groups over time in physical activity levels (or other obstetric health outcomes such as weight gain/retention, glucose control, and blood pressure), which is expected given the small sample size. However, all data demonstrate promise. For example, Figure 2 shows moderate-to-vigorous physical activity levels (MVPA) for both groups based on objective accelerometer data. Table 2 contains all accelerometry data. While not statistically significant, a clear trend is observed as the mean estimated difference between groups (when adjusted for BMI) changes from −0.35 (SE: 1.75) in mid-pregnancy to −0.81 (SE:1.75) in late pregnancy. Analyses were adjusted for BMI because BMI is associated with MVPA levels in the present study (midpregnancy: r = −0.410, *p* = 0.016) and in the existing literature [48]). There are clear and directionally consistent changes in MVPA; however, the large standard errors induced by our small sample size did now allow for statistical significance.

**Figure 3.**
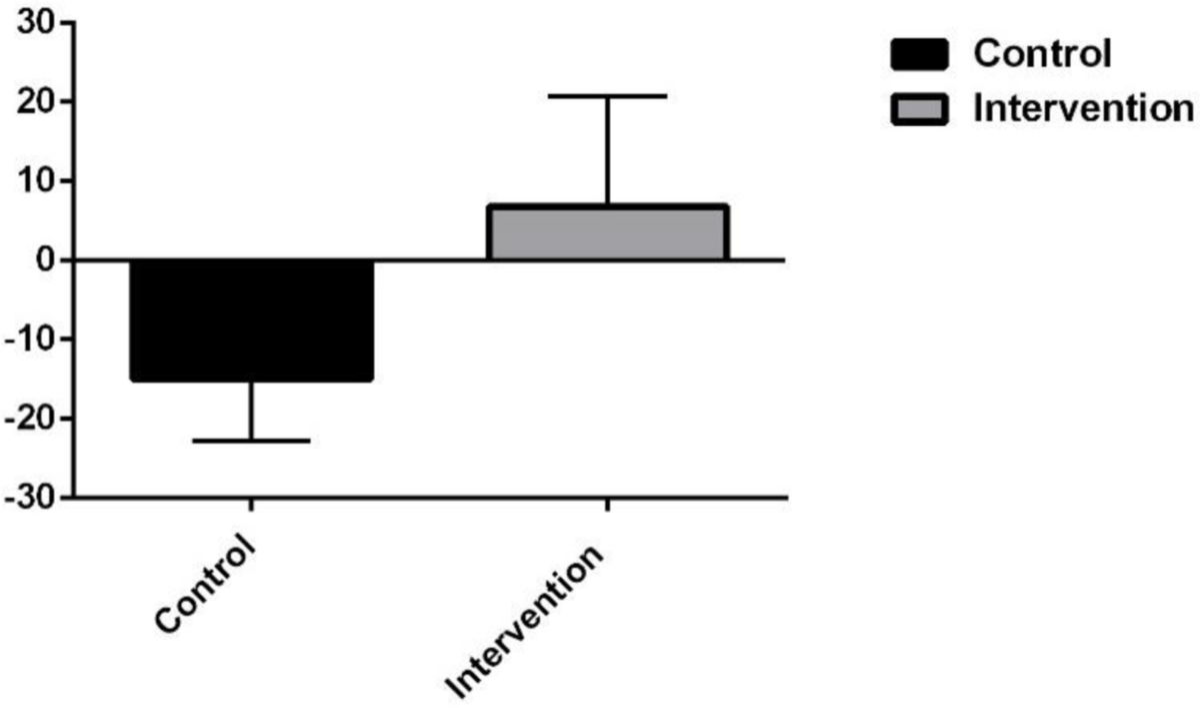
Change in total activity from mid-to-late pregnancy in control and intervention partic-pants (mean ± SEM).

**Figure 4.**
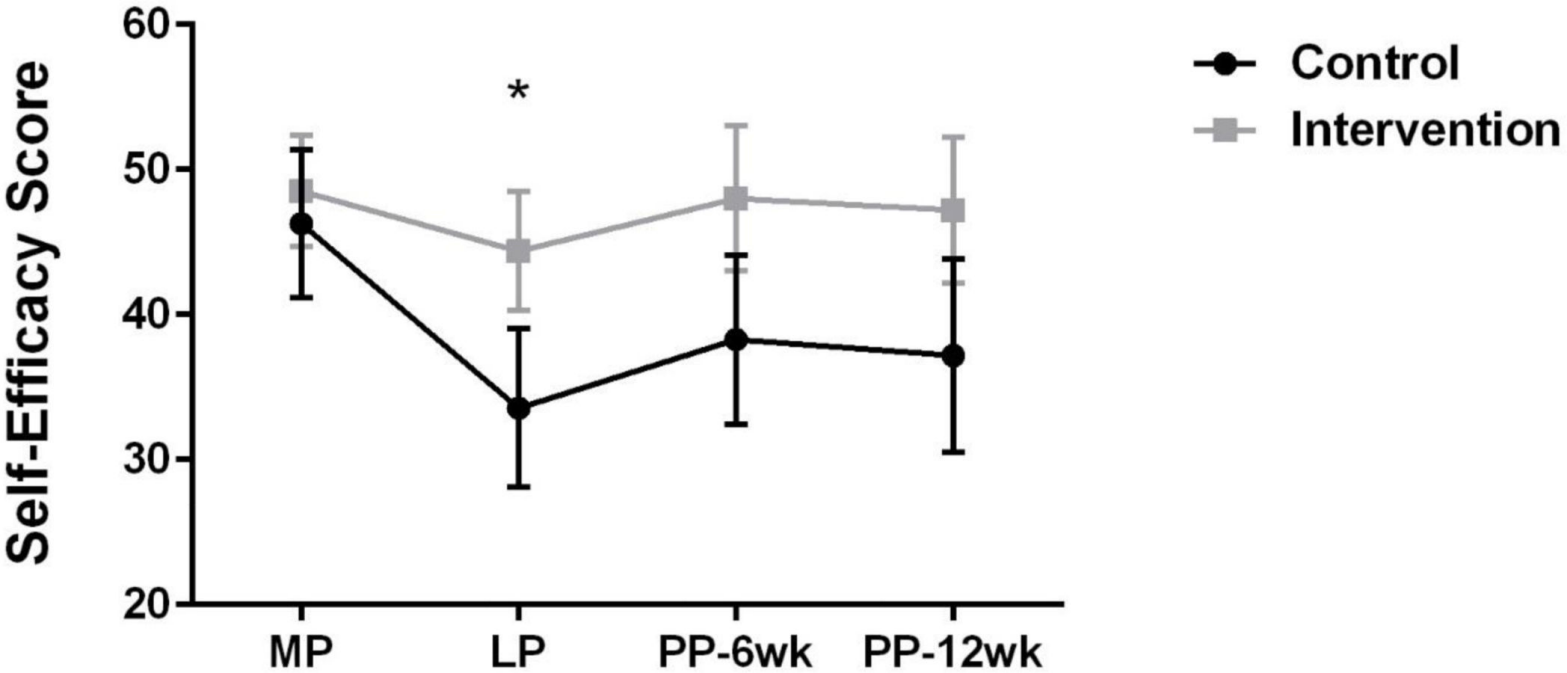
Self-efficacy foe exercise scores in control and intervention participants (mean ± SEM). * *p* < 0.05.

Figure 3 shows PPAQ data, demonstrating that the control group saw a 15.1% decrease in total activity (sum of light, moderate, and vigorous), while the intervention group still managed to increase their activity levels by 6.7% from mid to late pregnancy (*p* = 0.11) (Figure 4).

Self-efficacy (via the SEES) was assessed across all four timepoints and controlling for BMI, and the estimated difference of means (control-intervention) changed from 0.96 (SE: 6.53) at baseline to −7.64 (SE: 6.66) in late pregnancy and remained at −6.41 (SE: 6.79) and −6.70 (SE: 6.96) at 6 and 12 weeks postpartum, respectively. Of note, BMI was controlled for because BMI is correlated with self-efficacy for exercise both in the literature [49] and in the current study (late pregnancy and SEES: r = −0.412, *p* = 0.021). When assessing the change in SEES from basline to late pregnacy only, there was a statistically significant difference in self-efficacy between groups (*p* = 0.044) (Figure 4). Furthermore, self-efficacy scores (both groups) were correlated with physical activity (MVPA) (r = 0.421, *p* = 0.012).

Based on an app satisfaction survey (using the MARS, Table 3), participants found the app to have trustworthy (4.0 out of 5) and reliable (3.9 out of 5) information, and they identified the workouts as safe (4.2 out of 5). Analyses for additional clinical outcomes were assessed, but statistical significance was not obtained and these data are not reported.

## 4. Discussion

Compared to an attention control group, pregnant and postpartum women using BumptUp^®^ showed improved self-efficacy for physical activity. Thus, while the modest sample size precluded statistical significance for MVPA, we conclude that BumptUp^®^ has potential to increase physical activity behaviors among pregnant and postpartum women. Study findings suggest key refinements to be made per pilot project feedback and a larger, adequately powered clinical trial is warranted.

The increase in self-efficacy for exercise among intervention participants is a critically important first step as public health theories demonstrate self-efficacy is intricately linked to engagement in physical activity [30], and this is especially true among pregnant women [31,32]. This change in self-efficacy demonstrates the potential of well-designed mobile apps to positively influence physical activity-related self-efficacy and subsequent behaviors (i.e., physical activity levels). This result is further demonstrated by the relationship noted between physical activity and self-efficacy among women in the study. Given that most women experience a decline in physical activity as pregnancy progresses [50] due to many new barriers that exist [51], and that self-efficacy for activity late in pregnancy influences exercise levels [52], this significant result is important and suggests that BumptUp^®^ may favorably influence activity levels among pregnant women.

Important theoretical implications for this work exist. Because the features of the app were designed within the framework of the Health Belief Model [18], future intervention strategies should consider using the constructs of the Health Belief Model to guide the development of the intervention tools employed. Self-efficacy was added to the Health Belief Model because it can better explain individual differences in health behaviors [53,54]. Given the known relationship between self-efficacy for exercise and exercise behavior [55], targeting pregnant and postpartum women’s ability to feel confident in their ability to be active is critically important and is oftentimes ignored in exercise interventions. It is important to design interventions that not only seek to provide physical support for exercise, but mental support as well. Interestingly, self-efficacy for exercise may be even more important for determining long-term health outcomes than self-assessed physical activity levels [51]. Women with higher levels of self-efficacy for exercise may have higher levels of general confidence, positive well-being, as well as reduced fatigue and psychological distress during and after exercise compared to those with low self-efficacy [56].

While post-test data did not demonstrate a statistically significant change in physical activity levels, these pilot data show promise given improvement in self-efficacy and other promising trends in the physical activity data. Given that physical activity levels typically drop as pregnancy progresses [50], a resource that can prevent or minimize this drop in activity levels has strong potential to favorably impact clinical outcomes, which is precisely what the data demonstrate in this pilot project.

At the end of the study, intervention participants were all contacted for an optional exit interview. Of the 19 contacted, 10 were interviewed. Women were given the opportunity to provide open-ended feedback regarding the app, which was overall very positive. For example, a representative quote from one pilot study participant at 14 weeks postpartum said:

“The BumptUp^®^ App acted as a great guide both during and after pregnancy. The activity tracker kept me accountable and motivated especially during the last few weeks before giving birth. The app provided excellent tips and educational articles that helped me make my way through the changes I was experiencing each week as well. After giving birth, the app didn’t stop! It was a great tool for a time when I needed support the most. The guided exercises may have been my favorite part because I knew that I would not be overexerting myself as I eased back into moving on a daily basis! Each workout was feasible yet challenging all at the same time, and I would recommend this type of guidance to any mom recovering from birth!”

The study team also asked each participant about the acceptability of the intervention itself, and all 10 reported that the surveys were not overly time-consuming and were well worth the small financial incentive. A common theme that emerged during the interviews was the appreciation of the electronic delivery of surveys so they could complete them anywhere/anytime as well as the porch delivery/pick-up of activity monitors. Feasibility and acceptability were demonstrated by the fact that only three women dropped out of the study (8% attrition rate), which is a strength of the study; interventions typically average a much higher rate of drop out [57]. Feasibility was also demonstrated by the fact that the sample was recruited in less than 9 months despite passive recruitment tactics (i.e., flyers hung at local clinics and on social media platforms from which potential participants reached out regarding participation).

In addition, the app satisfaction survey (the MARS tool [45]) provided feedback on areas to continue to develop and improve, which is a future direction of work. These include enhancing the capacity for customizations, improving usability and ease of navigation, and increasing the choices for workout programming within the app. Collectively, the information gathered from all of these data collections support the potential for impact of BumptUp^®^ in future trials.

While data from the BumptUp ^®^ pilot study did not demonstrate statistically significant improvements in physical activity levels (which was expected in a small pilot study), the app shows considerable promise for favorably impacting self-efficacy for exercise via the Self-Efficacy for Exercise Survey (SEES), and the importance of maintaining self-efficacy for exercise during a time point where physiologic changes can make exercise especially challenging for women [51] (i.e., pregnancy and recovering for pregnancy) should not be undersold. From a practical standpoint, the study is an important first step in showing that digital health technology can become an important and timely way to reach pregnant and postpartum women with much-needed physical activity education and support.

Limitations of the study include a small sample size; thus, statistical significance was unlikely to be obtained for many outcomes. In addition, the study population was not very diverse, thus limiting generalizability. Another limitation is the inability to directly determine adherence; the study team was unable to determine (based on app data) who achieved the goal of 150 minutes per week and logged this on the app. This is an important concept for future app improvement—an administrative feature that allows the team to determine who logged 150 min/week into the app and who did not. Despite the limitations, the study has many notable strengths. One strength of the study is its inclusion of an intervention designed based on public health theory and directly by key stakeholders. To our knowledge, BumptUp^®^ is the only app created that fits these criteria. Another strength is the objective quality of the data collections, particularly the physical activity data (Actigraph) and the medical outcome data directly from patient charts (not self-reported). Another notable strength is the low rate of attrition; most interventions have a higher rate of drop-out [57]. Another key strength is that the intervention proposed has potential for scalability. Many times, physical activity interventions are successful at changing outcomes, but the ability to scale them up and/or sustain them is impossible (i.e., an exercise intervention with one-on-one coaching sessions). BumptUp^®^ offers a potential solution that could easily be used by millions of women without placing burden on the health care team.

Given the maternal mortality and morbidity crisis in the United States, evidence-based and potentially sustainable intervention strategies are needed. The mobile app tested in this pilot project has potential to be further refined and scaled-up to serve women across the nation. Future directions include app refinement, a larger clinical trial, and additional testing among health disparity populations in order to make sure the app is effective, accessible and culturally appropriate for all women. Another pilot study trial is planned in a lower-middle-income country to test the acceptability and effectiveness of the app in another country. From a broader perspective, the opportunity that exists for BumptUp^®^ (and other digital health interventions) is unique and timely. Obstetricians are seeking resources to help patients improve health outcomes such as blood pressure, insulin resistance, complicated deliveries, and depression/anxiety; physical activity can improve all of these things. A simple resource that providers can give to patients, without increasing clinical demands or expecting them to prescribe exercise (for which they are not qualified [51]) is critical in our nation and world. With further testing and refinement, BumptUp^®^ could become that resource for pregnant women, postpartum women, and their healthcare team.

## 5. Patents

BumptUp^®^ is registered for US Trademark (Reg. No. 6,837,038; Serial No. 90–812,606, Registered 6 September 2022).

## Figures and Tables

**Table 1.** Demographic Information for Control and Intervention Participants.

	Control (n = 18)	Intervention (n = 17)	*p*-Value
Pre-pregnancy BMI (kg/m^2^)	30.2 ± 8.7	27.8 ± 6.0	0.348

Married	18 (100%)	16 (94%)	0.486

Annual income			
*>$20 k*	0 (0.0%)	2 (11.8%)	
*$20–40 k*	3 (16.7%)	3 (17.6%)	0.285
*$60–80 k*	3 (16.7%)	5 (29.4%)	
*>$80 k*	12 (66.7%)	7 (41.2%)	

Race			
*Caucasian*	18(100%)	17 (100%)	0.472

Educational Attainment			
*Highschool/GED*	1 (5.6%)	0 (0.0%)	
*Trade/Technical School*	0 (0.0%)	2 (11.8%)	
*Associates degree*	1 (5.6%)	0 (0.0%)	0.537
*Bachelor’s degree*	9 (50.0%)	9 (52.9%)	
*Master’s degree*	5 (27.8%)	4 (23.5%)	
*Doctoral degree or higher*	2 (11.1%)	2 (11.8%)	

Employed	16 (88.9%)	14 (83.4%)	

Self-reported health status			
*Excellent*	4 (22.2%)	8 (47.1%)	
*Very Good*	10 (55.6%)	7 (41.2%)	
*Good*	3 (16.7%)	2 (11.8%)	0.386
*Fair*	1 (5.6%)	0 (0.0%)	
*Poor*	0 (0.0%)	0 (0.0%)	

Baseline Physical Activity Levels (%)			
*Sedentary*	52.1 ± 14.7	51.8 ± 8.8	0.946
*Light*	35.3 ± 10.5	35.6 ± 6.5	0.932
*Moderate*	12.5 ± 5.9	13.5 ± 5.1	0.612

**Table 2.** Accelerometry data for all cut points across all participants at each time point of the study.

% of Time over 7 Consecutive Days (mean ± SD)									

		Sedentary			Light			Moderate	

Timepoint	IG	CG	All	IG	CG	All	IG	CG	All
Mid-Pregnancy	51.8 ± 8.8	52.1 ± 14.7	52.0 ± 12.1	35.6 ± 6.5	35.3 ± 10.5	35.4 ± 8.6	13.5 ± 5.1	12.5 ± 5.9	13.0 ± 5.5
Late Pregnancy	51.9 ± 10.9	53 ± 11.3	52.5 ± 10.9	34.3 ± 7.0	34.7 ± 7.4	34.5 ± 7.1	13.8 ± 5.7	12.3 ± 4.8	13.0 ± 5.2
12 weeks postpartum	48.8 ± 7.6	49.0 ± 8.0	48.9 ± 7.7	36.3 ± 5.4	37.3 ± 4.7	36.8 ± 5.0	14.6 ± 5.5	13.8 ± 5.0	14.3 ± 5.2

IG: Intervention Group; CG: Control Group.

**Table 3.** MARS survey data.

Question	Likert Scale Choices	Score (0–5)
How trustworthy is the information on the app?	Not trustworthy at all to very trustworthy	4.0 ± 0.9
How reliable was the information provided on the app?	Not at all to extremely reliable	3.9 ± 2.0
Was there enough information on the app?	Not enough to as much as I could want	3.5 ± 1.3
Was there enough education topics on the app?	Not enough to as much as I could want	3.5 ± 1.1
How would you rate the usability of the app?	Impossible to use to very easy to use	2.9 ± 1.1
How easy to use was the app?	Too confusing to use to very easy to use	3.0 ± 1.1
Did the app work well?	Impossible to use to functioned perfectly	2.9 ± 1.0
Was it easy to navigate the app?	Not at all to very easy	3.3 ± 1.1
Were there any problems with the app’s performance?	Too many to use to none at all	3.1 ± 1.3
How would you rate the quality of the workouts?	Very poor to excellent	3.4 ± 1.1
Did the workouts feel unsafe to you?	All of them to none of them	4.2 ± 0.9
Were the workouts fun?	Not at all to very fun	2.8 ± 1.1
Were the workouts an appropriate length for you?	Too long/short to perfect length	3.2 ± 1.0
Were you able to personalize the workouts as much as you wanted to?	Not at all to as much as I wanted to	2.6 ± 0.8
Was the app fun to use?	Not at all to very fun	2.9 ± 0.8
Were you able to tailor the app to you as much as you wanted?	Not at all to all I wanted	3.4 ± 1.0
Would you recommend this app to friends?	Would discourage use to would highly recommend	3.0 ± 1.0
What would your star rating of this app be?	1 through 5	3.3 ± 1.0
How likely are you to download this app and use it again?	Absolutely will not to absolutely will	2.8 ± 1.2

## Data Availability

The datasets used and/or analyzed during the current study are available from the corresponding author on reasonable request.
